# Acceptability and experiences of real-time continuous glucose monitoring in adults with type 2 diabetes using insulin: a qualitative study

**DOI:** 10.1007/s40200-024-01403-9

**Published:** 2024-03-05

**Authors:** Oscar T. Sergel-Stringer, Benjamin J. Wheeler, Sara E. Styles, Alisa Boucsein, Claire S. Lever, Ryan G. Paul, Rachael Sampson, Antony Watson, Martin I. de Bock

**Affiliations:** 1 Department of Women’s and Children’s Health, Dunedin School of Medicine, University of Otago, 201 Great King Street, Dunedin, 9016 Aotearoa New Zealand; 2 Department of Paediatrics, Te Whatu Ora Southern, Dunedin, Aotearoa New Zealand; 3 Department of Human Nutrition, Division of Sciences, University of Otago, 70 Union Street West, Dunedin, 9016 Aotearoa New Zealand; 4 Waikato Regional Diabetes Service, Te Whatu Ora, Hamilton, Aotearoa New Zealand; 5 Te Huataki Waiora, School of Health, University of Waikato, TT Building Hillcrest Road, Hamilton, 3240 Aotearoa New Zealand; 6 Aotearoa Diabetes Collective, 170 Collingwood Street, Waikato, Hamilton, 3204 Aotearoa New Zealand; 7 Department of Paediatrics, University of Otago, 4 Oxford Terrace, Christchurch, 8024 Aotearoa New Zealand; 8 Department of Paediatrics, Te Whatu Ora Waitaha Canterbury, Christchurch, Aotearoa New Zealand; 9 University of Otago, 2 Riccarton Avenue, Christchurch, 8011 Aotearoa New Zealand; 10 Department of Paediatrics, University of Otago, Christchurch, 8140 Aotearoa New Zealand

**Keywords:** Real-time continuous glucose monitoring, Self-monitoring of blood glucose, Experiences, Glucose monitoring

## Abstract

**Aims:**

To explore the lived experiences of initiating real-time continuous glucose monitoring (rt-CGM) use in individuals with type 2 diabetes using insulin.

**Methods:**

Twelve semi-structured interviews were conducted amongst individuals with type 2 diabetes taking insulin who were enrolled in the 2GO-CGM randomised controlled trial and had completed 3 months of rtCGM. Interviews were transcribed verbatim and analysed to identify common themes regarding their experiences.

**Results:**

The interviews revealed three key themes: i) rtCGM as a facilitator of improved health behaviours; ii) the acceptability of rtCGM systems compared to capillary blood glucose testing; and iii) barriers to the continual usage of rtCGM technology – including: connection difficulties, longevity of the sensors, and local cutaneous reactions to the sensor adhesive.

**Conclusion:**

Adults on insulin with type 2 diabetes find rtCGM systems widely acceptable, and easier to engage with than traditional self-monitoring of capillary blood glucose.

**Supplementary Information:**

The online version contains supplementary material available at 10.1007/s40200-024-01403-9.

## Introduction

Diabetes is a major global health concern affecting approximately 536.6 million people between the ages of 20–79 globally, with a global prevalence of 10.5% [1]. Estimates suggest 90% of these individuals have type 2 diabetes. Guidelines recommend glycaemic control, as measured by HbA1c, should be < 53 mmol/mol or 7% to mitigate possible complications [2–4]. However, only a minority meet these targets, highlighting that attaining this level of control remains challenging for most individuals [5].

Effective glucose monitoring is an important aspect of the management of those with type 2 diabetes on insulin therapy [2, 3]. Qualitative studies show several barriers to self-monitoring of blood glucose (SMBG), including frustration with readings of hyperglycaemia, needle-related fear, inconvenience, and a lack of self-efficacy [6]. Comparatively, real-time continuous glucose monitoring (rtCGM) systems provide continuous data at regular intervals to the user, thus can promote increased engagement and reduce the burdens associated with SMBG, however, cost barriers for rtCGM are well known. Recent studies have shown favourable outcomes in comparison to SMBG through greater reductions in HbA_1_C levels [7–11], as well as a reduction in hospital admissions for hypoglycaemia [12]. Furthermore, one study suggests that there may be a more significant improvement in HbA_1_C and diabetes distress for those with type 2 diabetes compared to those with type 1 diabetes [13].

Despite the potential benefits, limited research exists that explores the experiences of individuals with type 2 diabetes using modern rtCGM systems [8, 14, 15]. Existing research is mostly related to the use of intermittently scanned continuous glucose monitoring (isCGM), which are distinct from rtCGM as scanning is required to access glycaemic data. Thus, further qualitative research is crucial to understand the factors influencing rtCGM use and its impact on health and well-being to inform clinical education and support.

Therefore, this qualitative study delves into the experience of individuals with type 2 diabetes wearing a modern rtCGM device, emphasizing the acceptability and feasibility of use, as well as the impact on self-care behaviours.

## Methods

### Participants

Adults with type 2 diabetes, enrolled in the 2GO-CGM study, were first provided an information sheet outlining the research’s purpose. Following this, they were invited to complete informed consent for participation in this qualitative study.

In brief, the primary randomized controlled trial (RCT), conducted in Aotearoa New Zealand, specifically in Otago, Canterbury, and the Waikato, investigated the effect of initiating Dexcom G6 real-time continuous glucose monitoring (rtCGM) (Dexcom, Inc, San Diego, California) on glycaemic outcomes in adults with type 2 diabetes on at least one daily insulin injection. The inclusion and exclusion criteria for the 2GO-CGM study can be found in Supplementary Table 1, and a concise overview of the trial is available in Supplementary Table 2 [16]. The 3-month RCT was completed by 67 participants. Twelve participants who had been randomized to the rtCGM intervention were invited to participate in the qualitative study, and were identified through convenience sampling.

### Data collection

Between July 2022 – January 2023, semi-structured qualitative interviews were conducted, with only interviewers and participants present, over Zoom video conferencing software (Zoom Video Communications, Inc., San Jose, California). Topics included the acceptability and usability of the rtCGM sensor system, with specific emphasis on the individual experience of wearing the sensor, participant perspective on glycaemic control, and the impact of the system on health behaviours. Additionally, participants discussed barriers and facilitators related to sensor usage.

These interviews followed an open questioning technique with a brief non-pilot-tested interview guide (Supplementary Table 3). The interviews were predominantly conducted by OSS, a non-clinical member of the 2GO-CGM study research team with training and experience in conducting qualitative interviews. Some participants in the study (42%) had a pre-existing administrative relationship with OSS due to his role in the research team within their locality. Additionally, CL, a diabetes nurse specialist who was also a member of the 2GO-CGM research team, was present for one interview.

All interviews were recorded, transcribed verbatim, checked for accuracy, and de-identified. The interviews varied in length spanning 45 – 75 min, and no repeat interviews were conducted. Participants were not given the opportunity to review their transcripts. To provide additional demographic context, information such as age, gender, ethnicity, socio-economic status, clinical values (including HbA_`1_C levels prior to initiation of rtCGM), and the duration of diabetes diagnosis were retrieved from clinical records to describe the sample.

### Data analysis

The general inductive analysis of transcripts, carried out by OSS, was facilitated by NVIVO12 software (QSR International Pty Ltd, Melbourne, Australia) to apply codes to sections of text relevant to the research aims [17]. A pragmatic approach to coding was taken, rather than using a theory to guides the analysis, due to the research team valuing the application of the study findings in the clinical setting. The transcripts were read line-by-line and then meaningful segments were labelled and groups of codes were subsequently added to relevant categories. Code frequencies were reviewed to identify summary categories that captured the key aspects of themes identified in the transcripts. Subsequent interrogation with members of the research team elicited a consensus on the key themes. Thematic saturation was determined by when no new themes appeared in the final three interviews.

### Ethics

Ethical approval for this project was granted by the New Zealand Central Health and Disability Ethics Committee (ethics reference 21/CEN/75/AM01), in addition to consultation and approval of this study for Māori (the indigenous people of New Zealand) was provided by the Ngāi Tahu Research Consultation Committee. All participants gave informed consent to the interview, and the study was carried out in accordance with the Declaration of Helsinki.

## Results

### Participant description

Twenty-three participants of the 2GO-CGM study initially consented to participate; thereafter, twelve (52% of invited participants) were interviewed. Two people declined, three people were lost-to-follow-up following an interview booking, and six did not respond to further efforts to contact them. Most participants were women (73%), of New Zealand European ethnicity (91%), and had not previously used a form of CGM (82%). There was no discernible difference in demographics between the individuals who elected to participate in the interview and those who did not. Further information pertaining to the participants who participated is presented in Table 1.

**Table 1:** Demographics and clinical information of participants

Characteristics	Participants (*n* = *12*)
Age (years), median [range]	55 [37 – 64]
Gender, *n (%)*	
Female	8 (67)
Male	4 (33)
Prioritized ethnicity, *n (%)*	
New Zealand European	10 (83)
Māori	2 (17)
Socioeconomic deprivation index^*a*^, median [range]	5.5 [1–10]
Duration of diabetes (years), median [range]	15.5 [8–25]
HbA1c (mmol/mol), median [range]	
Prior to rtCGM	73 [44 – 109]
HbA1c (%), median [range]	
Prior to rtCGM	8.8 [6.2 – 12.1]
Insulin regimen prior to rtCGM^*b*^, *n (%)*	
Basal insulin only	3 (25)
Premixed insulin	1 (8)
Basal and bolus insulin	8 (67)
Daily frequency of SMBG prior to rtCGM, median [range]	2.5 [0 – 4.8]
Previous usage of CGM devices^*c*^, none *(%)*	9 (81)

*rtCGM* real-time continuous glucose monitoring, *isCGM* intermittently scanned continuous glucose monitoring, *SMBG* self-monitoring of blood glucose. CGM, continuous glucose monitoring. ^a^Calculated by The New Zealand Deprivation Index (2018) which provides deciles of socioeconomic status calculated using established data from home addresses with 1 representing the least deprived and 10 being the most deprived [18].^b^All participants were on a basal/bolus routine after 3 months using rtCGM. ^c^Those two who has used CGM previously had used isCGM devices

### Themes

The following three themes were identified: i) rtCGM systems as a facilitator of improved health behaviours; ii) the acceptability of rtCGM systems compared to capillary blood glucose testing; and iii) the barriers to the continual usage of rtCGM. Representative quotes for themes and sub-themes can be found in Table 2.

**Table 2:** Summary of themes, sub-themes, and representative quotes

Themes (n)	Quotations (participant no.)
rtCGM as a facilitator of improved health behaviours	
Improved sense of being ‘in control’ (12/12)	*1. “I guess if you think of it as, before the CGM, diabetes was like this little gremlin in my life that I had no control over and it would just throw things at me and I felt like I spent my life reacting to situations that’s diabetes impacted… Whereas, now I feel in control. I still have this gremlin in my life, but he’s housetrained, he’s not dictating” (Participant 1)*
Increased frequency of monitoring (11/12)	*2. “When I was on the manual fingerpricking it got to the point where I wasn't checking all that often because of the pain factor on my fingers; and now, I check it all the time…. Normally, 90% of the time we have our phones with us so it’s easy just to click open the app and see what your blood sugar’s doing” (Participant 3)*
Improved glycaemia (11/12)	*3. “Just halving my Hba1c, I’d never ever thought that would happen – ever. That has really astounded me. I’m sure, long term, my kidney function is not going to be altered, hopefully any damage I’ve had to my eyes won’t progress so far. So, all those long-term benefits that I’m yet to see as well” (Participant 9)*
	*4. “So I’ve just done my diabetes check [at the diabetes clinic service] and they were very, very pleased with my results. They haven’t been like that for a very, very long time” (Participant 11)*
Increased understanding of impact of behaviour on glucose levels (12/12)	*5. “Those graphs give me a good opportunity to see when my blood sugar’s do go up, or when they are going down. Also seeing, you know, the little thing going, ‘your blood sugars have been in range for, let’s say, 83% of the time’. That’s been brilliant because then I can look back and go ‘I’m doing what I need to do’” (Participant 2)*
	*6. “It may have just been yesterday when I saw the benefits of that [the rtCGM graph and trends]. We had a barbeque with family and I knew desert was coming out. So I’m looking at it [the rtCGM data] and I could see where the arrows were heading and I thought ‘that’s not gonna work’… So I picked up some desert and brought it back for later when I was in a better state” (Participant 12)*
	*7.“Sometimes if I look at it around lunchtime and my sugar levels are still quite high, I think I won’t bother with lunch. I’ll just wait a couple of hours. So, sort of looking at it to see if you actually do need to have something to eat” (Participant 7)*
Improved adherence to medication (10/12)	8*. “Now I always carry my insulin pen with me, and I use it more reactively. So, although I try to take it before each meal, I’ve also got it with me for, if any reason, my blood sugars are trending up so I can adjust my insulin during that period to make sure that it doesn’t go out of range. I never would have dreamt of taking my insulin out with me before [the trial]” (Participant 9)*
Improved sleep (6/12)	*9. “Going to sleep at night and not having to wake up at like 3 in the morning and prick your finger to find out if you are low or high. I actually have a monitor that wakes me if I have a low at three in the morning” (Participant 8)*
Improved mood (7/12)	*10. “It [the sensor system] has made my life easier. Before the sensor, my mental health was pretty bad. Whereas now it’s getting better, and my health seems to be getting better” (Participant 2)*
Engagement of others in diabetes-related care (9/12)	*11. “I’ve got my girlfriend linked into me [sharing rtCGM data]. So, I get phone calls if it’s [glucose levels] going up… If I get a high result we often sit and talk about what I had [eaten], and what to do next time” (Participant 5)*
Impact on work-life (6/12)	*12. “I was at work before I started [the rtCGM sensor system] and I’ve ended up shaky and couldn’t think… and I’ve gone home to bed. When I had the CGM I sort of felt I was heading in that direction, and I looked at it and it hadn’t gone off yet – but it was heading that way. So, I was able to go and find some sugar and it didn’t get to the point where I couldn’t focus” (Participant 4)*
Impact on social life (4/12)	*13. “I’m not finding excuses not to do things anymore because my blood sugars are too high. I’m not finding excuses not to do things because my sugars are too low. I can moderate my sugars properly. I can have the freedom to go wherever I want to go if I take my phone with me, which I have to do anyway” (Participant 8)*
Acceptability of rtCGM system usage compared to capillary blood glucose testing	
Ease of use (12/12)	*14. “This [the rtCGM sensor system] is much easier, it’s much better [than SMBG] … It’s like comparing a three-wheeled stagecoach with a Ferrari. It’s just revolutionary” (Participant 5)*
	*15. “Everything is so easy because I mean even when you change your sensor, your phone give you the directions as you do it. And if you turn it off [end the sensor session early], up comes all the instructions on how to change your sensor and what you need to do; even down to wiping it with the alcohol wipe” (Participant 8)*
Reduced time-burden (9/12)	*16. “It [SMBG] was a bit of a burden… It took so much time out of my day. Waking up, the first thing I’m having to do is prick my finger. Before I eat, having to prick my finger. After I’ve eaten, having to prick my finger. It was inconvenient, especially if I was out and about, having to find somewhere discreet enough to go and prick my finger” (Participant 2)*
Reduced invasiveness (11/12)	*17. “You don’t have the pain [when using the sensor] because it hurts – the fingerpricks. So, you don’t have that pain. But also, I worry about getting infections from pricking it because we use our fingers for everything” (Participant 4)*
Barriers to the continual use of rtCGM technology	
Technological difficulties (12/12)	18*. “It was like, I’ve put a new sensor in, I’ve put a new transmitter in, ‘What the hell? Why is it not working?’. I almost went through, actually I did go through, a full box of sensors just trying to get this one transmitter to bloody work” (Participant 2)*
	19*. “Every time I change a sensor, I go through every step the phone [rtCGM app] tells me to go through. I did it exactly the same way, but the sensor would not talk to the phone; it kept giving me an error message” (Participant 6)*
	*20. “Clarity [rtCGM data app] is not so good because quite often you have to uninstall Clarity and reinstall it. It doesn’t like the passwords and the actual sign-in codes quite often doesn’t work… So, it’s been a bit annoying” (Participant 3)*
Premature sensor loss (7/12)	*21. “I think there was one [premature sensor loss] where my bra strap caught it when I was taking my bra off. There was another where I knocked it [the sensor] off within a door when I caught it on the edge of a door. 98% of the time it [the premature sensor loss] would be due to the loss of stickiness [adhesive]” (Participant 1)*
Appearance of aged sensors (4/12)	*22. “I would like for the material around the sensor site to be waterproof…If it was waterproof, I just feel that it wouldn’t get as tatty looking. So, you know, being in a professional role, I get a bit self-conscious when it gets to this point, and I’ve got these rough edges and sometimes they get a bit grubby if I’ve been wearing a blacktop or something the previous day” (Participant 1)*
Plastic wastage (8/12)	*23. “It’s wasteful though, as in, all that plastic waste. It would be nice if it was more environmentally friendly” (Participant 7)*
	*24. “I think the packaging and the waste involved in the sensors is a bit of a shame really. I often feel quite guilty for putting all of that in the rubbish” (Participant 9)*
Bleeding or bruising at insertion site (6/12)	*25. “There’s been of blood on one occasion [when inserting the sensor]; not enough for me to worry about. I think it was just the way it went in or I might have scratched myself through the night and upset things” (Participant 10)*

i) rtCGM as a facilitator of improved health behaviours

Compared to SMBG, all participants reported increased engagement with diabetes management with rtCGM, as well as a positive impact on their health.

Prior to rtCGM, a vast majority of participants reported that their diabetes felt ‘out of control’. However, with rtCGM, all participants reported an increased sense of control, subsequently translating to improved self-efficacy in terms of treatment (quote 1). This had resulted in a myriad of perceived improved health behaviours including an increased frequency of glucose monitoring, improved glycaemia, more appropriate and adherent usage of medications, as well as self-directed dietary change. The added input from the specialist diabetes team, provided throughout this study, was reported by several participants to help develop these positive behaviours.

Almost all of the participants reported that they had increased their frequency of glucose monitoring (quote 2), mainly due to the perceived new-found ease of monitoring, the removal of the previous pain burden of finger-pricking, and an increased sense of being able to positively impact their glycaemia. Most participants reported seeing an improvement in their glucose levels, both in fewer episodes and severity of hyperglycaemia, and/or a decrease in laboratory confirmed HbA_1_C levels which all participants were provided with after 3 months using rtCGM (quotes 3 and 4). However, the majority of participants did note that the frequency of hypoglycaemia remained constant or increased, which may have been related to optimisation of diabetes medications and a concerted effort from the research team to lower glucose levels throughout the 2GO-CGM trial. However, the severity of hypoglycaemia was reported to have been better managed by rtCGM glucose alerts.

The real-time data provided advantages in the magnitude of information, as well as the on-going generation of graphs and trends in their glucose levels. Most participants noted this visualisation improved their management as they were able to observe and deduce, in real time, the impact of certain health-related decisions on their glycaemia (quote 5). As a result, participants reported an increased understanding of the impact of their diet on their glucose levels. Previously such engagement would have been more difficult with SMBG providing discrete data; specifically, nine participants made self-directed changes to their diets based on patterns observed with rtCGM (quote 6 and 7). Many of the participants also reported that rtCGM had resulted in improved adherence to all their medication. In terms of insulin, participants outlined that this was being taken more regularly and in more appropriate doses according to the trends they observed through rtCGM (quote 8).

rtCGM was also reported to have impacts on other aspects of health. Six participants reported that rtCGM had improved their sleep (quote 9). A further seven reported that their mood had become more stable (quote 10). rtCGM also was reported to facilitate the involvement of others within the participants’ diabetes care. Most participants reported that others became more involved with assisting with response to alarms for dysglycaemia (quote 11). The rtCGM sensors also functioned as a health-related conversation starter. Most of the participants reported this with a myriad of topics arising related to diabetes health including the impact of diet and mental health on diabetes. Five participants reported that they had already recommended the system, to others with diabetes, and every participant mentioned that they may recommend the system to others in the future.

Overall, this improved self-efficacy had an impact on work and social lives. Six participants reported that the system had positively impacted their capacity to fulfil work responsibilities through reduced episodes of symptomatic dysglycaemia, less time-burden with glucose monitoring, and increased sense of safety for those in potentially hazardous work environments (quote 12). Socially, four participants reported an increased willingness to attend social occasions with friends and loved ones due to reduced diabetes-related burden with rtCGM use (quote 13).

ii) Acceptability of rtCGM system usage compared to capillary blood glucose testing

Every participant reported that they preferred to use the rtCGM sensor system compared to SMBG using a capillary glucose meter. Firstly, every participant reported that these rtCGM systems were easier to use compared to SMBG (quotes 14 and 15). Multiple reasons were reported for this including the automation of the system, the simplicity of the user-experience, the accessible nature of the glucose levels, and access to online videos and tools to assist with trouble-shooting issues such as replacing a sensor. One of the main benefits was the reduced time to record a glucose level result. Several participants made note that SMBG took a significant portion of time out of their day. In comparison, every participant reported how the increased accessibility of rtCGM information was more convenient (quote 16).

The reduced invasiveness of rtCGM was also reported by many as an advantage compared to SMBG. ‘Finger-pricking’ was noted by all participants to be painful, and an important driver for a reduced frequency of monitoring. In comparison, several participants noted that using the rtCGM sensor was comparably painless (quote 17). Three participants reported only a single occasion of pain with insertion over their entire time using the system. For some participants the reduced burden of finger-pricking came with benefits related to having fewer open wounds on the ends of the fingers; notably an increased ability to use their hands for hobbies such as sewing, and a perception of a decreased chance of infection.

iii) Barriers to the continual usage of rtCGM

All participants reported wanting to continue to use rtCGM. Despite this, every participant reported some barrier which may prevent them from doing so. These varied greatly between participants, with several different barriers arising throughout the interviews.

The most commonly reported difficulty in using this new technology arose with difficulties related to the performance of the sensors or app (quotes 18–20). Every participant reported some difficulty with the technology at some point; however, the issues varied greatly between individuals. Some of the issues experienced by participants included issues with the range of the transmitter, Bluetooth® connection failures to the apps associated with the system or receiver, issues downloading and accessing the apps, and poor accessibility on mobile devices for visually impaired individuals.

Another barrier to the use of rtCGM was premature sensor loss. Seven participants reported having experienced a sensor falling off before the full ten days of sensor life had elapsed (quote 21); however, for four of these individuals this had happened only once. For the other three this was a common experience whereby most sensors would not last the full ten days. Most commonly this was due to problems with the adhesive. Another concern was the appearance of the sensors as the sensor life elapsed, with a few reporting that towards the end of the ten-day-period the sensors/dressings were beginning to appear tattered which impacted their perceived personal appearance in a negative way (quote 22). The plastic wastage through the use od sensors was also considered a barrier; especially when considering the regular cycle of sensor application and removal (quotes 23 and 24).

Several participants reported some adverse cutaneous reaction with the adhesive, however only one reported any serious reaction to sensor application. It was more commonly reported that on some insertions of the sensor there was bleeding and bruising at the site of application (quote 25).

### The future cost barrier

All the participants noted that cost would be a future barrier to using rtCGM technology beyond the completion of the 2GOCGM trial (if requiring self-funding). Eleven participants noted that the cost associated would prohibit them from using this technology despite a significant desire to continue because of the benefits to their health and lifestyle. Related quotes can be found in Table 3.

**Table 3:** Quotations describing the future cost barrier to using rtCGM technology

Quotations (participant no.)
*“I know I can’t afford it [the rtCGM system]. There’s no way on my income, because I’m single, there’s no way I could afford to fund it… Once this trial is over, I won’t be able to afford to continue using it, which will be really sad” (Participant 4)*
*“With all due respect to the manufacturers, at $4,554 [New Zealand dollars] a year, that’s not in my budget… It doesn’t matter how good it is, it’s not in my price range” (Participant 6)*
*“I cannot afford it [the rtCGM system] … I would never be able to afford these without help. This would be a luxury item, even though it’s essential for my health” (Participant 8)*
*“As much as I think it’s [the rtCGM system] done wonders for me, and I’m very grateful for it, I think to factor that into your normal monthly bills – I just don’t think I could. On a normal wage that’s a big expense and it’s a bit of a luxury really” (Participant 9)*

## Discussion

This study has highlighted that individuals with type 2 diabetes using daily insulin prefer rtCGM over traditional SMBG due to perceived health benefits, the facilitation of increased engagement with diabetes-related care, and the general acceptability of use. Thus, this study has provided further nuance for the discussion of the role of these devices in the management of type 2 diabetes, and has helped to provide insight in a previously under-researched area.

Previous quantitative research has shown the statistically significant improvements these individuals can achieve with continuous glucose monitoring (CGM) technology [7–12], but there remains a paucity of qualitative evidence to support its feasibility in this large demographic. This small data set suggests that individuals with type 2 diabetes may have increased engagement with their diabetes and general health behaviours with the adoption of rtCGM. Notably, rtCGM was reported to increase convenience of monitoring through reduced invasiveness and general diabetes-related burden when compared to SMBG. Specifically, SMBG involves finger-pricking, which was reported to be a rather unpleasant and time-consuming procedure, all of which can be foregone when using rtCGM as an alternative.

The findings from our study are consistent with other research with comparable methodology. A larger survey-based study, predominantly isCGM based, supported our findings with results suggesting that 97% of individuals reported a positive health impact from their use of CGM technology [8]. Moreover, participants in this study reported that the devices provided meaningful information and were easy and comfortable to use [8]; our study for rtCGM also found this. Additionally a study exploring the impact of rtCGM in adolescents and young adults with type 2 diabetes, demonstrated that this technology had a beneficial impact of quality of life and the desire to enact positive behaviour change [14]. This mirrors the reports from our adult participants who reported better engaged through an increase in self-efficacy. Improvement in self-efficacy, especially in chronic health conditions, has been shown to significantly improve long-term outcomes in patients, including glycaemic control in those with type 2 diabetes [19, 20]. Our study shows that improved self-efficacy was often coupled with positive diet changes related to the feedback rtCGM provided on glycaemic response to specific foods. This is likely due to the capacity to visualise, track, and interpret specific changes as a result of the food they were consuming which provides increased understanding compared to what was previously offered by discrete measures by SMBG. This improvement in self-efficacy thereby increases their capacity to manage their condition through improved health literacy. Previous studies have also found this link, and suggest that that dietary modifications sustain beyond the conclusion of device usage [21, 22].

This dataset has also demonstrated that barriers still exist for the usage of rtCGM. Most commonly in this group this was associated with technology performance. This limitation may also have been worsened by difficulty adapting to new technologies due to a lack of familiarity, especially in individuals who have limited experience with smart phones and other similar technologies. Thereby user error may have contributed, however these devices still need to be feasible in these population thus our study has still revealed some critical information. Previous research describes the most significant barrier to prolonged CGM use as cutaneous complications, notably erythema, pruritus, and induration [23]. Only one individual in this dataset reported any cutaneous reaction resulting in brief cessation of use, however this was managed with topical steroid. Previous research supports the use of such steroids, and other barrier agents such as Cavilon (3 M, Saint Paul, Minnesota), to manage dermatological issues associated with the use of diabetes-related technologies [24]. Therefore, it appears that this major barrier may be able to be managed for most individuals, provided they have good access to additional supports.

The real-world context also limits the implementation of rtCGM technology. This trial provided devices free of charge; however, there remains a significant cost that has deterred many participants from this trial from continuing to use the system. The relevance of this finding is particularly relevant as type 2 diabetes is associated with a low socioeconomic position [8, 25, 26]. Therefore, any financial difficulties are likely to be exacerbated for these individuals. Cost-effectiveness analysis into CGM usage is generally favourable for this demographic for overall spending on health [27], however more research is required to assess whether funding is appropriate – especially as devices become more accessible [28].

The strengths of this study relate to the qualitative nature of its study design. Semi-structured interviews allowed for guided discussion led by the experience of the participants. While a sample size of 12 is a potential limitation in such a heterogeneous group, thematic saturation was achieved, thus we are confident that relevant themes were elucidated. The sample provided a diverse perspective due variable age, occupation, and locality of participants, however we acknowledge that the transferability of our results is limited due to local population demographics and health systems. Nevertheless, we believe that the transferability of these findings allows for these conclusions to be relevant for all those who are required to do SMBG for their diabetes management; beyond just those with type 2 diabetes. This is because our findings have shown a universal dislike for finger-pricking due to pain, discomfort, and time-burden; all of which are factors that cannot be erased without changing modality. Furthermore, our findings suggest, especially when compared to other studies in type 1 diabetes, that the feasibility of rtCGM is also universal with widespread consensus regarding the increased acceptability and feasibility in comparison to SMBG. An important limitation to acknowledge is the predominantly European sample with only two participants identifying as Māori. Thus, there may be some cultural barriers that remain unknown which may prevent some individuals from engaging with rtCGM. Additional limitations include the continuous interaction with a specialist diabetes team, and optimisation of medications, which may partially explain the glycaemic benefits experienced by participants. Convenience sample may have also biased the sample towards individuals engaging in more positive health behaviours despite suboptimal glycaemia control being a key RCT inclusion criteria. Moreover, this study may be impact by re-call bias as the questioning pertains to events over several months. Therefore, our results should be interpreted with some caution, and may not be generalisable to all people with T2D around the world.

## Conclusions

This study substantiates previous findings regarding rtCGM acceptability and barrier in adults with type 2 diabetes. It underscores the preference for rtCGM over traditional monitoring methods due to perceived benefits in glycaemic control, self-efficacy, and convenience. Nonetheless, challenges persist related to technology performance, environmental concerns, cutaneous complications, cost, and equity.

### Supplementary Information

Below is the link to the electronic supplementary material.

Supplementary file1 (DOCX 29 KB)

## Data Availability

The data that support the findings of this study are available on request from the corresponding author. This data is not publicly available due to potential privacy issues for the participants who took part.

## Abbreviations

rtCGM: Real-time continuous glucose monitoring
SMBG: Self-monitoring of blood glucose
isCGM: Intermittently-scanned continuous glucose monitoring
CGM: Continuous glucose monitoring
