# Supplementary material for: Acceptability and experiences of real-time continuous glucose monitoring in adults with type 2 diabetes using insulin: a qualitative study
Source: J Diabetes Metab Disord. 2024 Mar 5;23(1):1163–71. doi: 10.1007/s40200-024-01403-9 (PMC11196444; doi:10.1007/s40200-024-01403-9)
Supplement: Supplementary file 1 — Supplementary file1 (DOCX 29 KB) [file 40200_2024_1403_MOESM1_ESM.docx]

Supplementary Table 1: Inclusion and Exclusion Criteria for participation in the 2GO-CGM Study

| Inclusion Criteria | Exclusion Criteria |
| --- | --- |
| 1. Diagnosis of Type 2 diabetes*^a^* 2. HbA_1_C >8.0% or >64 mmol/mol*^b^* 3. Daily insulin requirement of ≥0.2 units of insulin/kg/day for >3 months 4. Aged ≥16 years 5. Be willing and able to conform to the study protocol | 1. Medical history of another form of diabetes such as T1DM, monogenic diabetes, secondary pancreatic diabetes, diabetes due to endocrinopathies 2. Hospital admission for hyperglycaemia in the previous 6 months 3. Use of systemic corticosteroids in previous 14 days, or repeated pharmacological systemic sources of corticosteroids 4. Recurrent of chronic systemic infections that significantly impact glycaemia*^c^* 5. Major cardiovascular event or major surgery in last 3 months*^d^* 6. Active malignancy requiring ongoing treatment 7. Previous or planner bariatric surgery 8. Pregnancy 9. Inability of the individual or legal guardian to provide informed consent 10. Any other reason that the investigator believes may not be in the best interest of the individual to participate |

*T1DM, Type 1 Diabetes Mellitus. MODY, Maturity-Onset Diabetes of the Young. ^a^As per American Diabetes Association (ADA) classification defined by the ADA Standards of Medical Care in Diabetes 2022 (29). ^b^Laboratory confirmed diagnosis in the previous 30 days. ^c^Determined on the discretion of the investigators. ^d^Major cardiovascular event includes, but is not limited to, myocardial infarctions, cerebrovascular accidents, coronary artery bypass grafts, or percutaneous transluminal coronary angioplasty.*

Supplementary Table 2: Overview of 2GO-CGM Trial

| 2GO-CGM Study Overview |
| --- |
| The 2GO-CGM is a multi-site 12-week randomised controlled study, followed by a 12-week continuation phase with those initially randomised to routine care cross over to the rtCGM intervention^a^. This main study is then followed by a 12-month extension. Both arms of the study will be treated with the same management algorithm throughout the 24-week study phase (excludes 2-week run-in period), and the participants will be contacted by research staff at week 2, 8, 14 and 20 to help titrate medications as indicated by the algorithm.  Expected duration of study participation is 26 weeks:   - Baseline Visit: Commence 2-week run-in period and baseline data collection (blinded rtCGM device for all participants) - Week 0 Visit: Randomisation (rtCGM intervention or routine care) - Week 10 Visit: Blinded rtCGM for control arm - Week 12 Visit: Primary end point, repeat baseline measures, intervention arm is eligible for qualitative interview - Week 24 Visit: Study completion, repeat baseline measures, control arm is eligible for qualitative interview   After completion of the main study, participants will be invited into the 12-month extension study   - 6-month Visit: Repeat baseline measures |

*rtCGM, real-time continuous glucose monitoring. Intervention is the Dexcom G6 real-time continuous glucose monitoring system (Dexcom, Inc, San Diego, California) with appropriate training on how to use the device*

Supplementary Table 3: Qualitative interview guide

| Main questions (with suggested discussion points) |
| --- |
| 1. To begin, can you tell me what made you interested in taking part in this study? |
| 1. Can you tell me about how you have found wearing the sensor? |
| Positives (e.g., reduced lows/highs)  Negatives (e.g., skin reactions)  Participant expectation vs. reality  Alerts |
| 1. Can you tell me what it was like changing from using a glucose meter & finger pricking to using a CGM sensor?  - Training experience (e.g., potential improvements) - Difficulty of the technology - Changes in participant glucose monitoring (e.g., frequency) - Easier or more difficult than finger-pricking - Any difference in understanding glucose reports/graphs |
| 1. Can you tell me about anything you found particularly useful with the sensor system?  - Alarms - Trend arrows - Less testing - More data - Sharing data |
| 1. Tell me about any difficulties or barriers you experiences to using the system?  - Alarms - Cutaneous reactions to adhesive - Premature sensor loss - Pain on insertion - Tech issues with app - User-friendliness of interface |
| 1. Can you tell me how the sensor changed the way you manage your health?  - New habit (individual and support network) - Changes to diet or exercise - Medication changes (e.g., insulin adjustments) - Changes to quality of life/mental health/diabetes distress/burden |
| 1. Could you tell me about whether you would recommend this Dexcom system to someone else with diabetes? Why/why not? |
| 1. Is there anything else you think we should know for the research? |
